# Young Children’s Motor Interference Is Influenced by Novel Group Membership

**DOI:** 10.3389/fpsyg.2016.00321

**Published:** 2016-03-08

**Authors:** Johanna E. van Schaik, Hinke M. Endedijk, Janny C. Stapel, Sabine Hunnius

**Affiliations:** Donders Institute for Brain, Cognition and Behaviour, Radboud University NijmegenNijmegen, Netherlands

**Keywords:** motor interference, social groups, copying behavior, early childhood, interpersonal coordination

## Abstract

From early childhood onward, individuals use behavior copying to communicate liking and belonging. This non-verbal signal of affiliation is especially relevant in the context of social groups and indeed both children and adults copy in-group more than out-group members. Given the societal importance of inter-group interactions, it is imperative to understand the mechanistic level at which group modulations of copying occur early in development. The current study was designed to investigate the effect of novel group membership on young children’s motor behavior during a simultaneous movement-observation and -execution task. Four- to six-year-olds (*n* = 65) first gained membership to one of two novel groups based on their color preference and put on a vest in their chosen color. Subsequently, they were instructed to draw a straight line back-and-forth on a tablet computer that was concurrently displaying a stimulus video in which a model moved her arm congruently or incongruently to the child’s instructed direction. In half of the stimulus videos the model belonged to the in-group, while in the other half the model belonged to the out-group, as identified by the color of her dress. The deviations into the uninstructed direction of the children’s drawings were quantified as a measure of how much observing the models’ behaviors interfered with executing their own behaviors. The motor interference effect, namely higher deviations in the incongruent trials than in the congruent trials, was found only for the out-group condition. An additional manipulation of whether the models’ arms followed a biological or non-biological velocity profile had little effect on children’s motor interference. The results are interpreted in the context of the explicit coordinative nature of the task as an effect of heightened attention toward interacting with an out-group member. This study demonstrates that already during early childhood, novel group membership dynamically influences behavior processing as a function of interaction context.

## Introduction

Copying the behaviors of others occurs in many forms and plays a fundamental role in early social-cognitive development (Jones, 2009; Over and Carpenter, 2013; Marshall and Meltzoff, 2014; Paulus, 2014). Imitative play guides toddlers’ everyday interactions with adults (Killen and Uzgiris, 1981) and peers (Nadel, 2002; Eckerman and Peterman, 2004). By the age of two, children’s copying behavior is sensitive to the social availability of an adult model (Nielsen, 2006; Nielsen et al., 2008; Nielsen and Blank, 2011). This social sensitivity increases during early childhood (Over and Carpenter, 2012), as early preferences for similar others (Fawcett and Markson, 2010; Mahajan and Wynn, 2012; Haun and Over, 2013) expand to encompass even arbitrary distinctions to demarcate groups (Dunham et al., 2011; Buttelmann and Böhm, 2014; Plötner et al., 2015). By the age of five, children mimic and imitate the behaviors of novel in-group members more than out-group members (Watson-Jones et al., 2016; van Schaik and Hunnius, under review) and children use information about who copies whom to infer interpersonal affiliations (Over and Carpenter, 2015). These social effects of copying are not confined to childhood; the bi-directionality between copying those you like and liking those who copy you plays an important, often implicit role in adulthood (Chartrand and Lakin, 2013; Lakin, 2013). Hence, throughout the lifespan, but already starting during early childhood interactions, behavior copying is an essential means of communicating similarity and belonging (Over and Carpenter, 2012; Heyes, 2013; Lakin and Chartrand, 2013).

Underlying behavior copying is a neurocognitive coupling between observing and executing actions (Molenberghs et al., 2009; Heyes, 2013; Paulus, 2014; Hamilton, 2015). Ontogenetically, this ‘mirror system’ is shaped through both observational and active experience (Hunnius and Bekkering, 2014), making it a dynamic product of an infant’s social environment (Heyes, 2010, 2013). Additionally, adult neuroimaging studies indicate that mirror system activation is modulated by social group membership. Mirror system and related activation triggered by the observation of actions has been found to be higher when the individual performing the action is an in-group member than an out-group group member, both for pre-existing and novel groups (Gutsell and Inzlicht, 2010; Molenberghs, 2013; Molenberghs et al., 2013; Rauchbauer et al., 2015).

However, the period in-between forming observation-execution associations during infancy and the mirror system’s social sensitivity in adulthood is understudied. During the preschool years, the complexity of the social environment in which young children execute and coordinate their behaviors expands and social groups increasingly play a role in daily interactions (Eckerman and Peterman, 2004; Rubin et al., 2006). Considering the social communicative function of copying behaviors reviewed above (Over and Carpenter, 2012), it is imperative to understand social, and particularly group, modulations of copying on a mechanistic level during early childhood.

The motor interference task (Kilner et al., 2003) provides a versatile behavioral measure of observation-execution associations and their modulators. This task, though importantly not a direct measure of neural mirror system activation, is based on the notion that if observing a behavior and executing a behavior elicit overlapping representations, then doing both simultaneously could cause interference (Kilner et al., 2003; Blakemore and Frith, 2005). In the original study, participants moved their arm back-and-forth in a straight line either horizontally or vertically while concurrently observing a confederate performing the same movement in the congruent or incongruent direction. As expected, in the incongruent trials, participants’ movement paths showed significant deviations into the direction of the uninstructed axis compared to both congruent trials and baseline trials without concurrent observation. Conditions with a robotic arm instead of a human confederate, though, elicited no interference in the participants’ movements, which the authors interpreted as an indication that the task is especially sensitive to biological movements (Kilner et al., 2003).

In a developmental adaptation of the task, Marshall et al. (2010) had 4-year-olds draw straight lines back-and-forth in either horizontal or vertical movements on a tablet computer screen using a stylus. At the same time, the screen was displaying a video of an adult female standing upright and moving her arm in either the congruent or incongruent direction. Like adults, the children in this study experienced motor interference (Marshall et al., 2010). As an initial exploration of the contribution of social factors on children’s motor interference, the experiment was then repeated with two different models. In a within-participants design, 4.5-year-olds performed the task atop stimuli of either a same-aged boy or an adult male. The children experienced interference in the peer condition but the interference effect for the adult model disappeared. The authors place the findings in the context of a “like me” framework, emphasizing the social relevance of similar individuals (Marshall et al., 2010). Yet, it is unclear whether the “like me” effects were driven by social factors, since the peer was a possible friend, or biological factors, since the participants’ own arm movements were more similar to the peer’s movements due to their similar body proportions. Thus, although laying the groundwork, this study’s results do not uniquely identify whether social factors influence young children’s motor interference.

A following developmental study investigated the influence of movement profile more closely (Saby et al., 2011). In a similar tablet version of the task, 4- to 5-year-old children drew atop a bear puppet moving with a biological or non-biological movement profile. The puppets had previously been animated or not during a story telling session. Contrary to expectations, though, motor interference was found for the biologically moving previously unanimated condition and non-biologically moving previously animated condition. The authors interpreted these results as an attentional effect of expectation violations that resulted from a mismatch between movement profile and animacy (Saby et al., 2011). Taken together, while these two developmental studies (i.e., Marshall et al., 2010; Saby et al., 2011) demonstrate the usability of the task with young children, the data are inconclusive as to the distinct influences of social and biological factors on children’s motor interference.

The current study was designed to investigate the influences of social and biological factors on young children’s motor interference more directly. Importantly, given the central role of social groups in young children’s copying behaviors as reviewed above, as well as the aforementioned evidence suggesting a specific influence of social groups on adults’ mirroring, we implemented a novel group manipulation. This provided a developmentally relevant manipulation and allowed us to measure the sensitivity of copying mechanisms to group processing effects without confounding the groups with past group experience or familiarity (Cikara and Van Bavel, 2014). By explicitly labeling group belonging and exposing the children to repeated interactions (i.e., trials) with in- and out-group models, the groups remained salient throughout the experiment. Additionally, by independently manipulating the movement profile (i.e., biological vs. non-biological) of in- and out-group models, we could isolate the influence of biological factors. Consequently, a 2 (congruency) × 2 (group membership) × 2 (movement profile) within-participants design was used.

It was expected that the motor interference effect would be replicated, by finding higher deviations into the uninstructed drawing direction in incongruent than congruent conditions. Also, interactions of both group membership and movement profile with congruency were expected. Observing in-group members was hypothesized to lead to greater interference effects than observing out-group members, in line with higher copying rates of in-group members than of out-group members (van Schaik and Hunnius, under review). Following the adult motor interference literature (Kilner et al., 2007), it was hypothesized that biological movements would lead to more interference than non-biological movements. Finally, an interaction between congruency, movement profile and group membership was expected in the direction of in-group biological trials showing the most interference.

## Materials and Methods

### Participants

Seventy children (35 female) participated at two primary schools in the Netherlands. Two children did not complete the experiment. The data of three children was at two or more standard deviations from the mean (see also Data Preparation) and was excluded from the final analyses. The final sample consisted of 65 4- to 6-year-olds (33 female; *M* = 63.85 months, *SD* = 7.27 months). Signed informed consent was acquired from the guardian prior to participation. The schools could choose their preferred type of compensation: one school opted for each child receiving a sticker post-participation and the other school opted for a book voucher for the classrooms. This research was approved by the local social science faculty’s ethical committee.

### Stimuli

**FIGURE 1:**
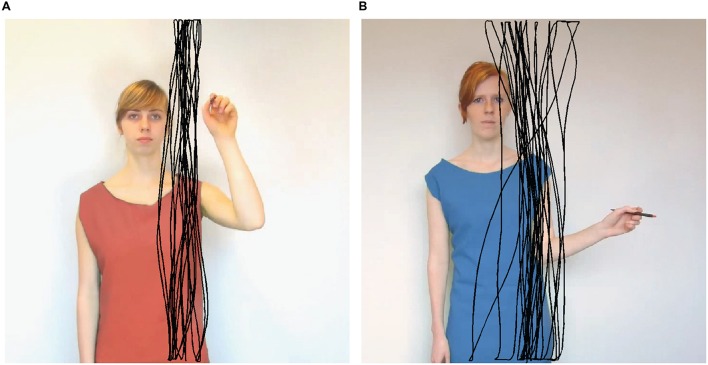
**Still frames of two stimulus videos illustrating different conditions.** An example participant’s drawings from a congruent **(A)** and incongruent **(B)** trial is overlaid in black. The stylus did not leave a line on the screen during the experiment.

Stimulus videos displayed one of three female models (a–c) from the waist up. The baseline stimulus (used for both the practice trials at the beginning of the experiment and baseline trials halfway through the experiment, see also Procedure) consisted of a video recording of model a wearing a green dress and standing still. In this manner, the baseline stimulus only differed from the experimental stimuli in the absence of arm movements, thus controlling for other factors such as body sway. In the experimental stimuli, the model (i.e., models b and c; **Figure 1**) was wearing a blue or red dress. The model moved her arm vertically or horizontally back and forth. The biological movement stimuli were recorded at 25 frames per second. Loops (consisting of one back-and-forth movement) were selected for their straightness and how well they matched the other model’s and directions’ (i.e., vertical and horizontal) speeds. These loops were then repeated back-to-back such that one stimulus video showed 10 repetitions of the loop (note: this was also done for the baseline stimulus with a segment of 1.5 s). The non-biological movement stimuli consisted of compiled photographs (frame rate = 25) in which the model’s arm did not follow a typical biological velocity profile of slowing down at the returning points. Instead, the model’s arm position shifted 10° between every two pictures, resulting in a triangular velocity profile. Stimulus videos lasted on average 16.6 s (range 15.9–18.0). The models’ dress colors (i.e., blue and red) were digitally edited, such that a full counterbalancing of model identity (i.e., models b and c) and color was possible.

Both stimulus display and the acquisition of data were performed with Presentation software (www.neurobs.com) on a tablet computer (Asus Eee Slate). The stimuli were cropped to be square (720 × 720 pixels; 146 mm × 146 mm on the tablet screen). A hard plastic sheet with an opening overlaying the area of the screen where the videos were played was placed over the tablet screen, to limit the area on which children could draw to precisely the square dimensions of the video. The stylus’ position on the screen was acquired at 100 Hz.

### Procedure

At the start of the experiment, the child was asked to draw a picture in Microsoft Paint in order to familiarize her with the stylus and tablet computer. Once the child had finished the drawing (or after 2 min), the experimenter started the experiment on the tablet computer. First, the colors red and blue appeared on the left and right sides of the screen (counterbalanced), and the child was asked to tap the stylus on the color they liked more (49% of the sample chose blue). The experimenter congratulated her on her choice and told her that she now belonged to that group. The child was given a vest to wear in the chosen color and the experimenter emphasized the group membership by exclaiming, “Wow! Now you are completely [chosen color], great!”

A practice session followed in which the baseline stimulus was shown twice, once as a horizontal practice and once as a vertical practice (order counterbalanced). The practice trials and all subsequent experimental and baseline trials followed the same procedure; before each stimulus, the screen was black while the experimenter instructed the child to draw a straight line back-and-forth either from side to side (horizontal) or top to bottom (vertical). The experimenter ensured that the child was holding the stylus at an appropriate starting position prior to starting the stimulus video (e.g., on the top or bottom of the screen for vertical trials, or at the left or right side of the screen for horizontal trials). Children were instructed to draw for the duration of each video (i.e., on average, 16.6 s of drawing per stimulus).

Following the two practice trials, the experimenter introduced the child to the two group models. A neutral picture of each model was shown for 7 s, accompanied by the experimenter’s explanation, “Look! She (also) belongs to the [color] group. She is (also) wearing [color] clothes.” The child was then informed that she would be seeing videos of these models and would have to draw lines like in the practice trials.

During the experimental trials, the experimenter instructed the child as for the practice trials; the experimenter instructed which direction to draw in and ensured the child held the stylus at an appropriate location while the screen was still black before each stimulus video started. The factors congruency (congruent vs. incongruent), movement profile (biological vs. non-biological), and group membership (in-group vs. out-group) were fully balanced within each child’s randomization of experimental trials (i.e., eight trials). Whereas direction drawn was counterbalanced within participants, direction observed was counterbalanced across participants; each child drew half of their trials horizontally and half vertically, but always saw either vertical or horizontal videos.^1^ Halfway through the experimental trials (i.e., after four trials), the child took a break from working on the tablet by playing a game of Memory for a few minutes. After the break, two baseline trials (i.e., one vertical and one horizontal; order counterbalanced) were performed using the baseline stimulus following the same procedure as the practice and experimental trials. This was followed by the remaining four experimental trials.

At the end, explicit preferences were measured by showing the neutral pictures of the two models on either side of the screen. The experimenter asked two questions in a randomized order (question 1: Who do you like more?; question 2: Who would you like to play with?) and the child responded by tapping the picture of the model she preferred. Before bringing the child back to the classroom, the experimenter thanked the child and emphasized that because the game was over, the groups no longer mattered.

### Data Preparation

Motor interference was measured per trial as the standard deviation of all the sampled locations where the screen was touched in the uninstructed axis throughout the trial (Marshall et al., 2010; Saby et al., 2011). To account for individual variability in drawing ability, this was divided by the same measure (i.e., the standard deviation in the uninstructed axis) from the corresponding (i.e., horizontal or vertical) baseline trial, resulting in a ‘deviation ratio’. Across participants, baseline outliers were first calculated per direction drawn at two or more standard deviations from the mean. Subsequently, outliers in the deviation ratios were calculated per condition per direction drawn also at two standard deviations. Outlying trials were excluded on a trial-by-trial basis and only three participants did not contribute any trials to the analyses.

## Results

First, the efficacy of the social group manipulation was tested. Explicit preferences were analyzed with a binomial test per question. The proportion of children who chose their in-group model in response to the question who they would like to play with (observed proportion = 0.70) was significantly higher than would be expected by chance (i.e., 0.50; *p* = 0.002). In response to the question regarding which model the children liked more, the proportion of children who chose their in-group model did not differ from chance (observed proportion = 0.54, *p* = 0.615). As a control, a chi-square analysis verified that the models were counterbalanced across participants in representing in- and out-group members (*p* > 0.250).

**FIGURE 2:**
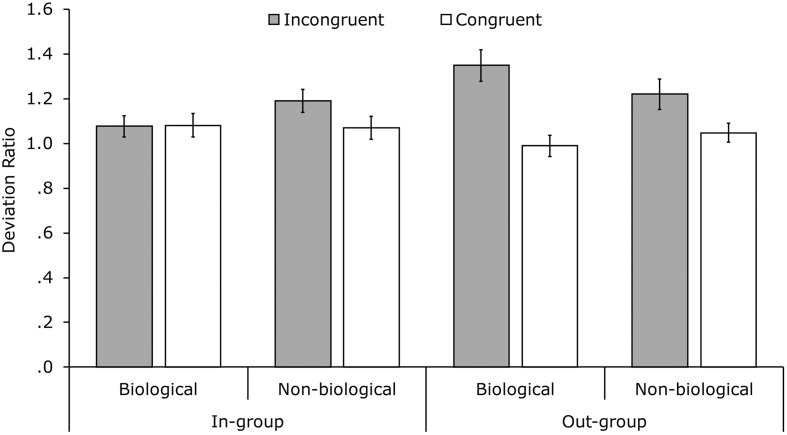
**Mean deviation ratios per condition.** Deviation ratios were calculated by dividing the standard deviation in the uninstructed direction by the standard deviation in the uninstructed direction from the corresponding baseline trial. Error bars indicate standard errors of the mean.

A linear fixed-effect model by means of maximum likelihood estimation was used. The model was performed on the deviation ratios with the factors congruency, group membership, and movement profile (full-factorial; **Figure 2**) and direction drawn as a covariate. There was a significant main effect of congruency, *F*(1,385.81) = 17.12, *p* <0.001, *r* = 0.21, with deviation ratios in the incongruent conditions (*M* = 1.21, *SE* = 0.031) being higher than in the congruent conditions (*M* = 1.05, *SE* = 0.024). No main effects of movement profile nor group membership were found. Conversely, there was a two-way interaction between congruency and group membership, *F*(1,389.36) = 7.24, *p* = 0.007, *r* = 0.14, and a three-way interaction between congruency, movement profile and group membership, *F*(1,396.72) = 4.10, *p* = 0.044, *r* = 0.10.

The interactions were tested further by repeating the analysis for the in-group and out-group conditions separately. For the in-group analysis there were no significant effects (congruency main effect: *p* = 0.143, all other *p*s > 0.250). The out-group analysis indicated a main effect of congruency, *F*(1,195.43) = 19.42, *p* < 0.001, *r* = 0.30. Deviation ratios for incongruent out-group trials (*M* = 1.28, *SE* = 0.049) were significantly higher than those for congruent out-group trials (*M* = 1.02, *SE* = 0.032). The interaction between congruency and movement profile did not reach significance (*p* = 0.175, *r* = 0.10). With respect to the original three-way interaction, **Figure 2** and the lack of any effects within the in-group suggest that this interaction was partially driven by the higher difference between incongruent and congruent trials in the biological out-group conditions (difference = 0.339) than in the non-biological out-group conditions (difference = 0.178). In sum, significant interference effects were found in the out-group condition but not in the in-group condition and no significant effects of movement profile were found.

## Discussion

In this study, the effects of novel group membership and movement profile on 4- to 6-year-olds’ motor interference were investigated. Participants performed back-and-forth movements either congruently or incongruently with respect to an in-group or out-group model’s movement direction. The expected motor interference effect was replicated, as incongruent conditions differed significantly from congruent conditions. This effect was only present for the out-group condition and did not occur for the in-group condition. Although there was also an interaction with movement profile, the effect of whether the models moved biologically or non-biologically was minimal.

An explicit measure indicated that the group allocation was effective in eliciting an in-group preference. Whereas the abstract question regarding liking did not show an in-group bias, the concrete question of whether children would like to play with the in-group or out-group model did show a significant in-group preference within the sample. This is in line with other studies using the same questions, in which the concrete question shows stronger effects with this age group (van Schaik and Hunnius, under review). This finding indicates that the out-group modulation of the interference effect, although opposite to expectation, holds bearing.

Initially, more motor interference was expected to occur for in-group members than for out-group members. Since motor interference is an effect of action observation-execution coupling, and this, in turn, is thought to contribute to behavior copying, it was expected that the motor interference would reflect the general finding that we (unintentionally) copy individuals we like more than individuals we like less (Chartrand and Lakin, 2013; van Schaik and Hunnius, under review). In favor of this underlying mechanism, a range of adult studies provide evidence for motor interference, under controlled circumstances, being a measure of action observation–execution coupling (e.g., Kilner et al., 2007) and for social modulations of mirror system activation (Molenberghs, 2013). However, in contrast to adult motor interference studies in which social factors are carefully controlled or discrete instances of mimicking an interaction partner’s behavior, the present continuous-action measure was embedded in an explicitly instructed social context. As a result, an additional overlaying process involving task-related social motivations likely influenced the underlying mechanisms, and hence influenced the behavioral effects more strongly. For instance, the explicit emphasis on the social groups and the continuous nature of the movements might have led children to experience the task as an instance of coordination (Richardson et al., 2009); like other instances of coordination such as dancing together, participants were carrying out a similar, continuous behavior in the same space as the models. And since interpersonal coordination is a means of establishing liking and affiliation between individuals (Hove and Risen, 2009; Lakin, 2013), additional social goals might have complicated the group manipulation’s effect. Here, the out-group motor interference might have been caused by heightened attention toward the out-group model as a result of a need to overcome intergroup differences in what might be experienced as an affiliative, spatially coordinative task.

Two recent studies have found analogous results to those of the present study. In a motor interference study, adults saw pro-social words (e.g., ‘group’) or anti-social words (e.g., ‘alone’) superimposed on the screen displaying the model. Contrary to expectation, the anti-social word condition led to higher motor interference than the pro-social condition. One of the authors’ interpretations of the findings holds that the anti-social condition threatened the “social harmony” of the interaction leading to increased attempts to affiliate with the model through increased coordination (Roberts et al., 2015, p. 7). Likewise, in a study using novel groups, adult participants who performed a repetitive rhythmic interaction with an out-group member spontaneously synchronized more than those who interacted with an in-group member. The authors similarly interpreted these findings as an effect of overcoming the inter-group differences, paralleling findings of synchrony being used to increase affiliation (Miles et al., 2011). In sum, the out-group interference effect observed in the present study might be a result of increased processing of the out-group member’s movement stemming from a desire to overcome the differing group memberships.

Notably, though, variants of this account could lead to the same effects. Group boundaries can be perceived as competitive, even in the absence of explicit competition (Cikara and Van Bavel, 2014). Hence, the inter-group differences that in a cooperative case lead to increased affiliation attempts as discussed above (Miles et al., 2011), in another case might lead to wanting to appease a threat through affiliation (Rauchbauer et al., 2015), or in yet a third, more distinct case, could lead to enhanced monitoring of a competitor to facilitate prediction of their potentially dangerous behavior (Gutsell and Inzlicht, 2013; Cikara and Van Bavel, 2014). Each case, though, would lead to increased processing of out-group movements. With respect to this study, one could argue that because children were brought into close contact with a potential threat (i.e., an out-group member), the enhanced interference effect was a result of increased vigilance of the out-group’s movement. However, this seems less likely for several related reasons. Threat effects in adults have primarily been found for existing groups and less so for novel group boundaries, which is likely caused by novel group manipulations leading to in-group preferences but not necessarily out-group derogation (Brewer, 1999; Cikara and Van Bavel, 2014). Also, explicit competition leads to considerably more intergroup hostility than simply dividing individuals into groups (Cikara and Van Bavel, 2014). Indeed, considering the age of the current study’s participants, these latter considerations are particularly relevant; novel-group-based out-group hate appears to develop between the ages of 6 and 8 (Buttelmann and Böhm, 2014), hence at a later age than the participants in the current study. Nonetheless, the out-group motor interference finding illustrates the complexity of social manipulations in combination with interpersonal tasks and indicates that this dynamic interplay of factors should be investigated further (Cikara and Van Bavel, 2014; Roberts et al., 2015).

Regarding the movement profile, only a limited effect on interference was found, and this was within the already-salient out-group condition. While past adult studies using a full-body paradigm have found higher interference in biological movement conditions (Kilner et al., 2007), a previous developmental study using a similar tablet-based design as the present study also found unexpected effects with respect to movement profile (Saby et al., 2011). Notably, in the tablet adaptation of the task used in this and past developmental studies, the similarity between the participants’ and models’ movements is reduced as compared to full-body paradigms. In the present stimuli (and in the past study’s puppet stimuli reported in Saby et al. (2011), though the puppets are anatomically less similar than the present study’s human models) the models make full shoulder-initiated arm movements that cross the midline as in the original adult paradigm but the participating children are asked to make unilateral movements with their wrists and hands in a precision pen-grip. As a result, the extent to which executing their action and observing the model’s action elicit overlapping representations is limited, hence reducing the motor interference effect. Our attempt to make wrist and hand configuration more similar between participant and model by having the model hold a pen in her hand seems to have produced insufficient overlap. Additionally, the aforementioned social task demands which led to increased saliency of the out-group condition, possibly diminished attention toward less salient features of the videos (i.e., the kinematic differences) reducing the overall influence of the movement profile manipulation even more. Yet, since within the out-group condition the pattern tended toward biological trials leading to more interference than non-biological trials, the interference that was measured is also not merely a spatial congruency effect. Taken together, the degree to which the observed action and the executed action overlapped, and the saliency of the different characteristics of the stimulus (e.g., social group vs. movement profile) likely diminished the extent to which the movement profile manipulation affected children’s motor interference.

## Conclusion

This study investigated the sensitivity of children’s motor interference to group membership and movement profile. Motor interference was only found for out-group members’ movements. This effect likely stems from heightened attention toward out-group members as a result of the coordinative nature of this explicitly instructed paradigm. Thus, this work demonstrates that the context of an interpersonal interaction uniquely interacts with the situation’s social dynamics, and consequently this interplay affects underlying imitative processes. Future research should continue to investigate how social factors affect copying mechanisms during early childhood, as it is crucial in understanding inter-group interactions.

## Author Contributions

The study was conceived of and designed by JS and JvS under the supervision of SH. The data were analyzed by HE, JvS, and JS. JvS drafted the manuscript and HE, JS, and SH provided extensive feedback. All authors approved the final version.

## Conflict of Interest Statement

The authors declare that the research was conducted in the absence of any commercial or financial relationships that could be construed as a potential conflict of interest.

## Footnotes

1 In a pilot, a full counterbalancing of models’ movement direction (i.e., 16 trials) proved too long in duration for this age group.

## References

[B1] Blakemore S. J. Frith C. (2005). The role of motor contagion in the prediction of action. *Neuropsychologia* 43 260–267. 10.1016/j.neuropsychologia.2004.11.012 15707910

[B2] Brewer M. B. (1999). The psychology of prejudice: ingroup love our outgroup hate? *J. Soc. Issues* 55 429–444. 10.1111/0022-4537.00126

[B3] Buttelmann D. Böhm R. (2014). The ontogeny of the motivation that underlies in-group bias. *Psychol. Sci.* 25 921–927. 10.1177/0956797613516802 24474724

[B4] Chartrand T. L. Lakin J. L. (2013). The antecedents and consequences of human behavioral mimicry. *Annu. Rev. Psychol.* 64 18.1–18.24. 10.1146/annurev-psych-113011-143754 23020640

[B5] Cikara M. Van Bavel J. J. (2014). The neuroscience of intergroup relations: an integrative review. *Perspect. Psychol. Sci.* 9 245–274. 10.1177/1745691614527464 26173262

[B6] Dunham Y. Baron A. S. Carey S. (2011). Consequences of “minimal” group affiliations in children. *Child Dev.* 82 793–811. 10.1111/j.1467-8624.2011.01577.x 21413937 PMC3513287

[B7] Eckerman C. O. Peterman K. (2004). “Peers and infant social/communicative development,” in *Blackwell Handbook of Infant Development*, eds Bremner G. Fogel A. (Oxford: Blackwell), 326–350. 10.1111/b.9780631212355.2004.00016.x

[B8] Fawcett C. A. Markson L. (2010). Similarity predicts liking in 3-year-old children. *J. Exp. Child Psychol.* 105 345–358. 10.1016/j.jecp.2009.12.002 20092828

[B9] Gutsell J. N. Inzlicht M. (2010). Empathy constrained: prejudice predicts reduced mental simulation of actions during observation of outgroups. *J. Exp. Soc. Psychol.* 46 841–845. 10.1016/j.jesp.2010.03.011

[B10] Gutsell J. N. Inzlicht M. (2013). “Using EEG mu-suppression to explore group biases in motor resonance,” in *Neuroscience of Prejudice*, eds Derks B. Scheepers D. Ellemers N. (London: Psychology Press), 278–298. 10.4324/9780203124635

[B11] Hamilton A. F. D. C. (2015). The neurocognitive mechanisms of imitation. *Curr. Opin. Behav. Sci.* 3 63–67. 10.1016/j.cobeha.2015.01.011

[B12] Haun D. Over H. (2013). “Like me: a homophily-based account of human culture,” in *Cultural Evolution: Society, Technology, Language, and Religion*, eds Richerson P. J. Christiansen M. H. (Cambridge, MA: MIT Press), 117–130. 10.1007/978-1-4939-1387-9_6

[B13] Heyes C. (2010). Where do mirror neurons come from? *Neurosci. Biobehav. Rev.* 34 575–583. 10.1016/j.neubiorev.2009.11.007 19914284

[B14] Heyes C. (2013). “What can imitation do for cooperation?,” in *Cooperation and its Evolution*, eds Sterenly K. Joyce R. Calcott B. Fraser B. (Cambridge: MIT Press), 313–331.

[B15] Hove M. J. Risen J. L. (2009). It’s all in the timing: interpersonal synchrony increases affiliation. *Soc. Cogn.* 27 949–960. 10.1521/soco.2009.27.6.949

[B16] Hunnius S. Bekkering H. (2014). What are you doing? How active and observational experience shape infants’ action understanding. *Philos. Trans. R. Soc. B Biol. Sci.* 369:20130490 10.1098/rstb.2013.0490 PMC4006192 24778386

[B17] Jones S. S. (2009). The development of imitation in infancy. *Philos. Trans. R. Soc. Lond. B Biol. Sci.* 364 2325–2335. 10.1098/rstb.2009.0045 19620104 PMC2865075

[B18] Killen M. Uzgiris I. C. (1981). Imitation of actions with objects: the role of social meaning. *J. Genet. Psychol.* 138 219–229. 10.1080/00221325.1981.10534136

[B19] Kilner J. M. Hamilton A. F. Blakemore S.-J. (2007). Interference effect of observed human movement on action is due to velocity profile of biological motion. *Soc. Neurosci.* 2 158–166. 10.1080/17470910701428190 18633814

[B20] Kilner J. M. Paulignan Y. Blakemore S. J. (2003). An interference effect of observed biological movement on action. *Curr. Biol.* 13 522–525. 10.1016/S0960-9822(03)00165-9 12646137

[B21] Lakin J. L. (2013). “Behavioral mimicry and interpersonal synchrony,” in *Nonverbal Communication*, 2nd Edn, eds Hall J. A. Knapp M. L. (Berlin: de Gruyter), 539–575.

[B22] Lakin J. L. Chartrand T. L. (2013). “Behavioral mimicry as an affiliative response to social exclusion,” in *The Oxford Handbook of Social Exclusion*, ed. DeWall C. N. (New York, NY: Oxford University Press), 10.1093/oxfordhb/9780195398700.013.0025

[B23] Mahajan N. Wynn K. (2012). Origins of “Us” versus “Them”: prelinguistic infants prefer similar others. *Cognition* 124 227–233. 10.1016/j.cognition.2012.05.003 22668879

[B24] Marshall P. J. Bouquet C. A. Thomas A. L. Shipley T. F. (2010). Motor contagion in young children: exploring social influences on perception – action coupling. *Neural Netw.* 23 1017–1025. 10.1016/j.neunet.2010.07.007 20732789

[B25] Marshall P. J. Meltzoff A. N. (2014). Neural mirroring mechanisms and imitation in human infants. *Philos. Trans. R. Soc. Lond. B Biol. Sci.* 369:20130620 10.1098/rstb.2013.0620 PMC4006193 24778387

[B26] Miles L. K. Lumsden J. Richardson M. J. Macrae C. N. (2011). Do birds of a feather move together? Group membership and behavioral synchrony. *Exp. Brain Res.* 211 495–503. 10.1007/s00221-011-2641-z 21448575

[B27] Molenberghs P. (2013). The neuroscience of in-group bias. *Neurosci. Biobehav. Rev.* 37 1530–1536. 10.1016/j.neubiorev.2013.06.002 23769813

[B28] Molenberghs P. Cunnington R. Mattingley J. B. (2009). ). Is the mirror neuron system involved in imitation? A short review and meta-analysis. *Neurosci. Biobehav. Rev.* 33 975–980. 10.1016/j.neubiorev.2009.03.010 19580913

[B29] Molenberghs P. Halász V. Mattingley J. B. Vanman E. J. Cunnington R. (2013). Seeing is believing: neural mechanisms of action-perception are biased by team membership. *Hum. Brain Mapp.* 34 2055–2068. 10.1002/hbm.22044 22290781 PMC6870530

[B30] Nadel J. (2002). “Imitation and imitation recognition: functional use in preverbal infants and nonverbal children with autism,” in *The Imitative Mind: Development, Evolution, and Brain Bases*, eds Meltzoff A. N. Prinz W. (Cambridge: Cambridge University Press), 42–62. 10.1017/CBO9780511489969.003

[B31] Nielsen M. (2006). Copying actions and copying outcomes: social learning through the second year. *Dev. Psychol.* 42 555–565. 10.1037/0012-1649.42.3.555 16756445

[B32] Nielsen M. Blank C. (2011). Imitation in young children: when who gets copied is more important than what gets copied. *Dev. Psychol.* 47 1050–1053. 10.1037/a0023866 21639617

[B33] Nielsen M. Simcock G. Jenkins L. (2008). The effect of social engagement on 24-month-olds’ imitation from live and televised models. *Dev. Sci.* 11 722–731. 10.1111/j.1467-7687.2008.00722.x 18801128

[B34] Over H. Carpenter M. (2012). Putting the social into social learning: explaining both selectivity and fidelity in children’s copying behavior. *J. Comp. Psychol.* 126 182–192. 10.1037/a0024555 21767011

[B35] Over H. Carpenter M. (2013). The social side of imitation. *Child Dev.* 7 6–11. 10.1111/cdep.12006

[B36] Over H. Carpenter M. (2015). Children infer affiliative and status relations from watching others imitate. *Dev. Sci.* 18 917–925. 10.1111/desc.12275 25529928

[B37] Paulus M. (2014). How and why do infants imitate? An ideomotor approach to social and imitative learning in infancy (and beyond). *Psychon. Bull. Rev.* 21 1139–1156. 10.3758/s13423-014-0598-1 24578090

[B38] Plötner M. Over H. Carpenter M. Tomasello M. (2015). The effects of collaboration and minimal-group membership on children’s prosocial behavior, liking, affiliation, and trust. *J. Exp. Child Psychol.* 139 161–173. 10.1016/j.jecp.2015.05.008 26112747

[B39] Rauchbauer B. Majdandžić J. Hummer A. Windischberger C. Lamm C. (2015). Distinct neural processes are engaged in the modulation of mimicry by social group-membership and emotional expressions. *Cortex* 70 49–67. 10.1016/j.cortex.2015.03.007 25929599

[B40] Richardson M. J. Campbell W. L. Schmidt R. C. (2009). Movement interference during action observation as emergent coordination. *Neurosci. Lett.* 449 117–122. 10.1016/j.neulet.2008.10.092 18996439

[B41] Roberts J. W. Bennett S. J. Hayes S. J. (2015). Top-down social modulation of interpersonal observation–execution. *Psychol. Res.* 10.1007/s00426-015-0666-9 [Epub ahead of print]. 25894232

[B42] Rubin K. H. Bukowski W. M. Parker J. G. (2006). “Peer interactions, relationships, and groups,” in *Handbook of Child Psychology*, 3rd Edn, ed. Eisenberg N. (Hoboken: John Wiley & Sons Inc), 571–645.

[B43] Saby J. N. Marshall P. J. Smythe R. Bouquet C. A. Comalli C. E. (2011). An investigation of the determinants of motor contagion in preschool children. *Acta Psychol.* 138 231–236. 10.1016/j.actpsy.2011.06.008 21783168

[B44] Watson-Jones R. E. Whitehouse H. Legare C. H. (2016). In-group ostracism increases high-fidelity imitation in early childhood. *Psychol. Sci.* 27 34–42. 10.1177/0956797615607205 26573906

